# Rabies postexposure prophylaxis in the United States: Opportunities to improve access, coordination, and delivery

**DOI:** 10.1371/journal.pntd.0009461

**Published:** 2021-07-15

**Authors:** Gavin T. Howington, Huy-Binh Nguyen, P. Brandon Bookstaver, Peter Akpunonu, Joshua T. Swan

**Affiliations:** 1 Department of Pharmacy Practice and Science, University of Kentucky College of Pharmacy, Lexington, Kentucky, United States of America; 2 Department of Pharmacy Services, University of Kentucky HealthCare, Lexington, Kentucky, United States of America; 3 Department of Medical Affairs, Kedrion Biopharma Inc., Fort Lee, New Jersey, United States of America; 4 Department of Clinical Pharmacy and Outcomes Sciences, University of South Carolina College of Pharmacy, Columbia, South Carolina, United States of America; 5 Department of Pharmacy Services, Prisma Health Midlands–Richland, Columbia, South Carolina, United States of America; 6 Department of Emergency Medicine, University of Kentucky HealthCare, Lexington, Kentucky, United States of America; 7 Kentucky Poison Control Center, Louisville, Kentucky, United States of America; 8 Department of Surgery, Houston Methodist, Houston, Texas, United States of America; 9 Department of Pharmacy, Houston Methodist, Houston, Texas, United States of America; Beijing Children’s Hospital, Capital Medical University, CHINA

## Introduction

Rabies remains a global public health concern that is responsible for more than 59,000 human deaths per year [1]. Postexposure prophylaxis (PEP) for humans exposed to rabies virus should include wound cleansing followed by 1 dose of human rabies immunoglobulin (HRIG) and a series of cell culture rabies vaccine doses [2]. Symptomatic rabies is nearly always fatal but universally preventable with appropriate PEP. An estimated 30,000 to 60,000 Americans receive rabies PEP each year [3]. The stakeholders involved in PEP implementation in the United States are diverse, creating systematic complexity that creates barriers to accessing care, lacks coordination, and delivers suboptimal care. Here, we elaborate on 3 issues of (i) access to, (ii) coordination of, and (iii) delivery of care for suspected rabies exposures; share examples of contemporary quality initiatives from large health systems; and propose novel solutions.

## Access to rabies PEP is limited and variable

Suspected rabies exposures are medical urgencies, which are serious but require relatively few interventions—thus, most patients presenting to the emergency department (ED) for rabies PEP are triaged to Emergency Severity Index level 4 (less urgent) or level 5 (nonurgent) [4]. Nevertheless, adequate and timely PEP is mandatory to prevent progression to fatal disease. Commonly, patients with wounds from an animal encounter will seek initial wound care in the ED and may be reasonably initiated on rabies PEP during the same ED encounter. Therefore, many EDs maintain inventory of rabies vaccine and HRIG. However, access to rabies PEP outside of the ED for less severe cases is limited. Sites that rarely care for patients requiring rabies PEP are not incentivized to maintain inventory of expensive rabies vaccine and HRIG. Payer reimbursement for clinic visits where rabies vaccine is administered have thin revenue margins and may lose revenue in some cases, which does not incentivize provider clinics to stock or administer rabies vaccine. Vaccine is infrequently covered by Medicare Part B or D plans and often results in high copays for patients. This limits patients’ ability to access follow-up vaccine doses, obligating them to seek follow-up vaccine doses in medical settings such as EDs. Typically, only pharmacies associated with travel clinics and EDs stock rabies vaccine, and retail pharmacies do not routinely stock HRIG. Due to these challenges in the US’ payer model, patients who seek rabies PEP outside of the ED setting may experience large out-of-pocket costs or have difficulty finding a care setting with available inventory in rural, suburban, and urban areas.

## Coordination of rabies PEP between care settings is lacking

**Fig 1:**
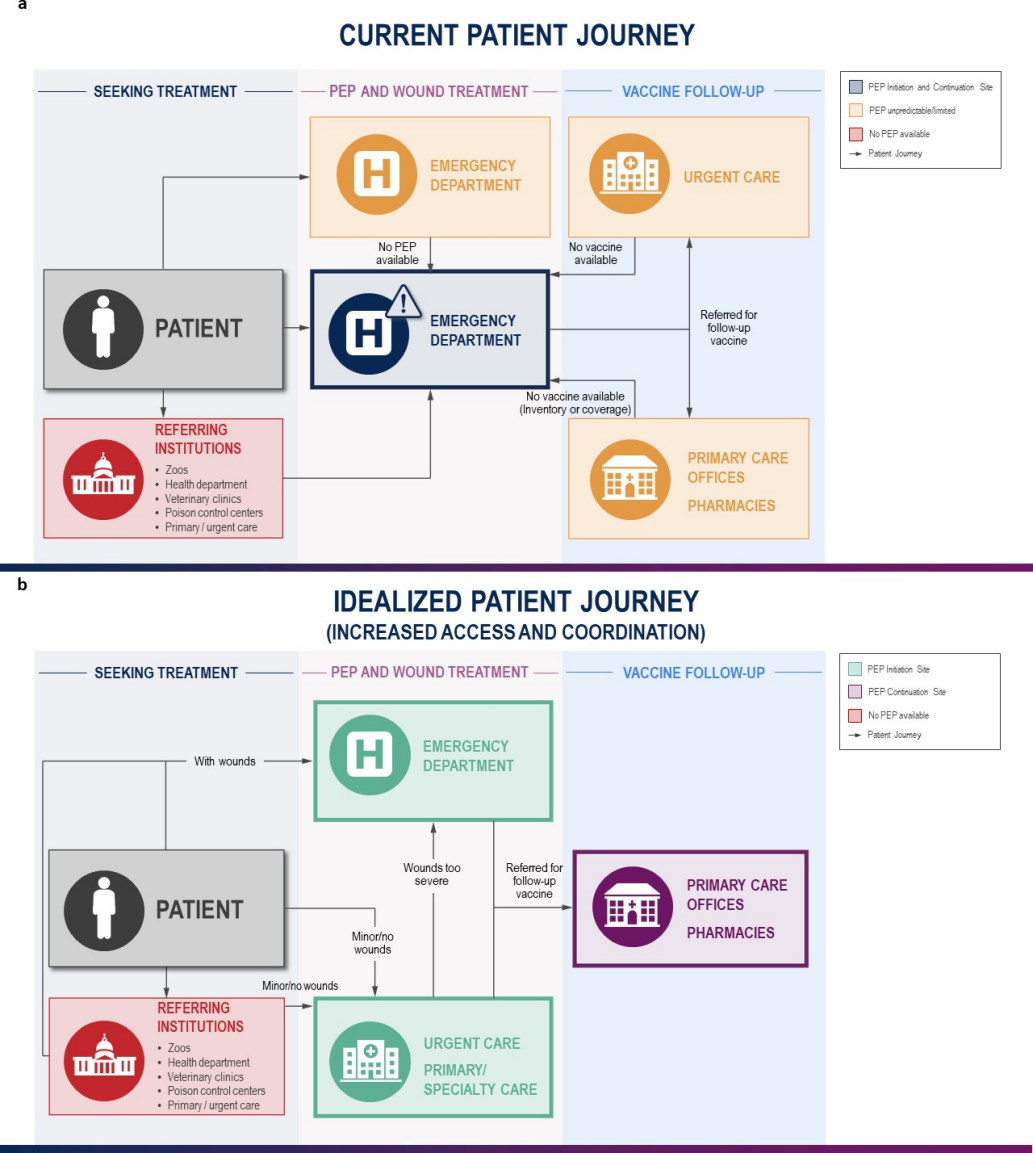
(**a)** Currently, patients with suspected rabies exposures routinely present or are referred to EDs regardless of whether they have extensive or minor/no wounds. Ambulatory patients may be referred by less acute healthcare settings and other stakeholders such as veterinarians, poison control and public health authorities, etc. Patients may be subsequently referred to community settings to complete PEP regimen of follow-up vaccine doses, but if community barriers to accessing care exist, patients will be referred back to the initiating ED. (**b)** Increasing access to PEP initiation in settings such as urgent, primary, and specialty care could be made possible by local availability or on-demand provision of HRIG. Patients with minor or no wounds could be adequately initiated on PEP at less acute settings, reducing burden on EDs. Availability of follow-up vaccination in community settings could further optimize the care chain and reduce burden on EDs. Boxes with a bold outline indicate a facility that can provide rabies PEP as a clinical service. ED, emergency department; HRIG, human rabies immunoglobulin; PEP, postexposure prophylaxis.

The US’ healthcare system lacks coordination in the chain of care for rabies PEP delivery that results in unnecessary ED visits, placing an undue burden on patients and payers. Since care settings outside of the ED provide limited access to HRIG, patients with minor wounds (or no wounds at all) are often referred to the ED to initiate rabies PEP (Fig 1A). In these cases, patients may endure a poor experience including long travel times and ED wait times, and ED resources are diverted from patients with more urgent complaints. When rabies PEP is initiated in the ED, staff are burdened with developing appropriate referral plans for subsequent rabies vaccine doses for patients with a variety of geographical and socioeconomic considerations. Unfortunately, due to patients’ inability to obtain rabies vaccine in the community, many unnecessarily return to the ED for subsequent rabies vaccine doses [5]. Wound management is the only aspect of rabies PEP that may require emergency care services. All other aspects of rabies PEP, including management of minor wounds, can be effectively provided outside of the ED.

## When given, delivery of rabies PEP is not optimal

As a result of access and coordination challenges in the US, EDs have become the *de facto* healthcare setting responsible for rabies PEP initiation. However, ED providers see proportionally fewer cases of rabies PEP per year compared to many other conditions. Additionally, the ED quality infrastructure has not developed extensive clinical decision support and referral systems for rabies PEP like they have for other diseases (stroke, acute coronary syndrome, trauma, etc.). Rates of correct and appropriate delivery of PEP (particularly HRIG) remain low, and many patients with anatomically feasible wounds may not receive HRIG wound infiltration in the ED [6,7]. Thus, an opportunity may exist to optimize implementation of care delivery and coordination.

Wound infiltration with as much HRIG as anatomically feasible to passively neutralize virus is crucial to bridge the protection gap between exposure and the active immune response to vaccination. Suboptimal delivery of rabies immunoglobulin, such as incomplete infiltration into wounds or intramuscular-only administration when a wound exists, may lead to rabies PEP treatment failure [8]. While guidelines agree on the importance of wound infiltration, neither the Centers for Disease Control and Prevention (CDC) nor the World Health Organization (WHO) define “anatomically feasible” volumes for infiltration by anatomical site. Similarly, HRIG uses weight-based dosing, which results in challenges for extremely small or large weights. Small bodyweight (i.e., children) may result in insufficient HRIG volume to optimally infiltrate large wounds. Large body weight (i.e., obese adults) may result in large volumes of HRIG that need to be delivered through many intramuscular injection sites. This lack of clarity may drive poor adherence and treatment gaps, including failure to infiltrate wound sites with HRIG.

## Examples of contemporary success from large health system perspectives

Although not comprehensive, several contemporary successes at large health systems aim to address some of these issues (Table 1). Several large institutions have implemented clinical decision support in their electronic medical records to optimize HRIG wound infiltration. The University of Kentucky HealthCare created a partnership with an affiliated urgent care to refer patients for subsequent doses of rabies vaccine and reduce unnecessary ED visits. The Houston Methodist health system utilized clinical decision support within their electronic health medical record to assist with rabies PEP medication selection and provide standardized administration instructions for HRIG. Additionally, Prisma Health Midlands created an outpatient pharmacy referral site within the health system for subsequent doses of rabies vaccine.

**Table 1:** Examples of rabies PEP quality programs in the US.

Health System (City, State)	Healthcare Settings Involved	Facilitating Access	Ensuring Coordination of Care	Improving Appropriate Delivery of Care
Houston Methodist (Houston, Texas)	1 academic medical center, 6 community hospitals, and 8 freestanding emergency care centers, approximately 380,000 visits/year	All EDs within health system provide HRIG and rabies vaccine	Patients are provided a flyer that lists pharmacies and clinics in metro area that can provide rabies vaccination	Developed an ED order set to assist with medication selection, dosing, and administration that emphasizes infiltrating eligible wounds with HRIG and avoiding administration of HRIG and rabies vaccine at same site
University of Kentucky HealthCare (Lexington, Kentucky)	1 academic medical center and 1 community hospital, approximately 129,500 visits/year.	All EDs provide HRIG and rabies vaccine	All rabies PEP patients receive a patient-specific flyer at ED discharge that details when to obtain days 3, 7, and 14 rabies vaccine doses and specifically instructs patients to schedule an appointment with the Urgent Care for subsequent rabies vaccine doses.	Developed an HRIG order with specific instructions describing wound infiltration, importance of avoiding administration of HRIG and rabies vaccine at same site, and recommending against mixing rabies vaccine with rabies immune globulin.
Prisma Health Richland (Columbia, South Carolina)	4 community hospitals, including 1 community, teaching hospital approximately 140,000 visits per year.	Majority of cases are handled in 1 ED within system, but urgent supply dose of HRIG is maintained at all locations	Patients are provided a hard copy of the vaccine series prescription in addition to the faxed or electronic copy to the outpatient pharmacy affiliated with the health system. The vaccine series prescription prompts the outpatient pharmacy to contact the patient if doses are not obtained on the appropriately scheduled timeline.	Developed an ED order set for both HRIG and rabies vaccine to facilitate continuity of the vaccine series at the affiliated outpatient pharmacy. This order prompts a faxed prescription to the outpatient pharmacy for adults or the Children’s Day Hospital for pediatrics.
		All EDs provide rabies vaccine		
			Local zoo staff and veterinarians direct patients to referral hospital in healthcare system	

ED, emergency department; HRIG, human rabies immunoglobulin; PEP, postexposure prophylaxis.

## Future directions

Flawed chain-of-care coordination leads to unnecessary treatment omission and financial burden on the healthcare system. Across the US, overabundant utilization of EDs for delivery of rabies PEP increases overall healthcare expenditure and significantly wastes ED resources. We identified barriers to implementing optimized referral systems based on acquisition costs of rabies vaccine and HRIG and lack of prescription insurance coverage of rabies vaccine by many payers. We also identified the need for guidance to clarify optimal HRIG administration in heterogeneous populations. We propose several imperatives to optimize patient care.

First, remove clinical and economic barriers to PEP access. As health systems validate effectiveness of clinical decision support built for ED providers, these tools should be broadly distributed to other EDs and adapted to provide clinical decision support in non-ED care settings, such as urgent care and primary care. Economic barriers can be mitigated either through reducing overhead and financial exposure associated with maintaining an inventory of rabies PEP or through enhanced reimbursement from payers for HRIG and vaccine. Strategies that reduce financial overhead from inventory include distributor consignment programs, product replacement programs, and “on-demand” delivery.

Second, create a healthcare coordination system for rabies PEP chain of care to reduce unnecessary ED visits for (1) initiation of rabies PEP when severe wounds or other urgent conditions are not present and (2) subsequent rabies vaccine doses. This could be accomplished through a publicly accessible registry of healthcare facilities (EDs, urgent care clinics, outpatient clinics, and pharmacies) that commit to providing either rabies vaccine or HRIG. Sites that provide both rabies vaccine and HRIG would be designated as “rabies PEP initiation sites,” and sites that provide only rabies vaccine would be designated as “rabies PEP continuation sites.” Rabies PEP initiation sites would need to ensure adequate staff training to determine which animal exposures require rabies PEP and to provide basic wound care, such as cleansing and bandages (Fig 1B). Patients could access this website to identify a site that provides initial healthcare and assessment following an animal exposure. This centralized resource could be leveraged by healthcare workers to design appropriate referral plans. Registries could be maintained and validated by agencies at the appropriate health department level.

Third, develop additional volume- and wound-based guidance for HRIG administration to enable appropriate assessment, triage, and closer adherence to authoritative guidelines. Guidance that describes optimal volumes based on locality and morphology of wounds could support practical implementation of CDC and WHO recommendations, particularly as guidelines move away from weight-based dosing [2,9,10]. For example, implementation of revised WHO guidelines in some European countries (e.g. in the Netherlands) have catalyzed a need for more granular guidance that describe minimum and maximum volumes of HRIG infiltration for each wound based on anatomical region [9,11].

Death from rabies is entirely preventable with appropriate care, which should be widely available. In the US, only 1 to 3 human rabies cases are reported annually, and death from rabies infection is rare [3]. However, systematic inefficiencies and barriers prevent patients from accessing appropriate and complete PEP, create unnecessary patient risk, and increase societal costs. Given the severe consequences from treatment failure and cost of therapy, rabies PEP should not be neglected by healthcare payers and referral systems in a developed country such as the US.

## References

[1] Hampson K, Coudeville L, Lembo T, Sambo M, Kieffer A, Attlan M, et al. Estimating the global burden of endemic canine rabies. PLoS Negl Trop Dis. 2015;9(4):e0003709. Epub 2015/04/17. doi: 10.1371/journal.pntd.0003709 ; PubMed Central PMCID: PMC4400070. 25881058 PMC4400070

[2] Manning SE, Rupprecht CE, Fishbein D, Hanlon CA, Lumlertdacha B, Guerra M, et al. Human rabies prevention—United States, 2008: recommendations of the Advisory Committee on Immunization Practices. MMWR Recomm Rep. 2008;57(RR-3):1–28. Epub 2008/05/23. . 18496505

[3] Centers for Disease Control and Prevention. Human Rabies [updated April 6, 2020; cited January 28, 2021]. Available from: https://www.cdc.gov/rabies/location/usa/surveillance/human_rabies.html.

[4] Agency for Healthcare Research and Quality. Emergency Severity Index (ESI): A Triage Tool for Emergency Departments Rockville, MD [updated May 20, 2020; cited January 28, 2021]. Available from: https://www.ahrq.gov/professionals/systems/hospital/esi.

[5] Iso T TA, Yuan F, Rizk E, Espino D, Nguyen NAA, Boyareddigari PR, Saldana RB, Swan JT. Characterization of unnecessary emergency department encounters for rabies postexposure prophylaxis vaccination. Value Health. 2020;23(S181):abstract 78.

[6] Hwang GS, Rizk E, Bui LN, Iso T, Sartain EI, Tran AT, et al. Adherence to guideline recommendations for human rabies immune globulin patient selection, dosing, timing, and anatomical site of administration in rabies postexposure prophylaxis. Hum Vaccin Immunother. 2020;16(1):51–60. Epub 2019/06/19. doi: 10.1080/21645515.2019.16326801632680. ; PubMed Central PMCID: PMC7012082. 31210569 PMC7012082

[7] Jerrard DA. The use of rabies immune globulin by emergency physicians. J Emerg Med 2004;27(1):15–9. Epub 2004/06/29. doi: 10.1016/j.jemermed.2004.02.005 . 15219298

[8] Wilde H. Failures of post-exposure rabies prophylaxis. Vaccine 2007;25(44):7605–9. Epub 2007/10/02. doi: 10.1016/j.vaccine.2007.08.054 . 17905484

[9] World Health Organization. Rabies Vaccines: WHO Position Paper. Wkly Epidemiol Rec. 2018;16(93):201–20. doi: 10.1016/j.vaccine.2018.06.061 30107991

[10] O’Brien KL, Nolan T, Rabies SWo. The WHO position on rabies immunization—2018 updates. Vaccine. 2019;37(Suppl 1):A85–A7. Epub 2018/10/22. doi: 10.1016/j.vaccine.2018.10.014 PubMed Central PMCID: PMC6863036. 30342901 PMC6863036

[11] Schreuder I, De Pijper C, van Kessel R, Visser L, van den Kerkhof H, Dutch advisory committee on r. Abandon of intramuscular administration of rabies immunoglobulin for post-exposure prophylaxis in the revised guidelines in the Netherlands in 2018: cost and volume savings. Euro Surveill. 2020;25(38). Epub 2020/09/26. doi: 10.2807/1560-7917.ES.2020.25.38.2000018 ; PubMed Central PMCID: PMC7533622. 32975188 PMC7533622

